# Influence of linear and angular parameters on lower third molar retention: the role of space allocation and transpalatal width

**DOI:** 10.3389/froh.2025.1612527

**Published:** 2025-09-22

**Authors:** Ana Isabel Contreras-Madrid, Roshan Melwani-Sadhwani, Rocío Trinidad Velázquez-Cayón, David Pérez-Jorge, Juliana Cassol Spanemberg

**Affiliations:** 1 Department of Dentistry, Faculty of Health Sciences, Fernando Pessoa Canarias University (UFPC), Gran Canaria, Spain; 2 University of La Laguna, Tenerife, Spain; 3 Department of Didactics and Educational Research, University of La Laguna (ULL) and National Distance Education University (UNED), Tenerife, Spain

**Keywords:** retained third molar, transpalatal width, available space, direction of eruption, prediction of third molar retention

## Abstract

**Background:**

The mandibular third molars are the most frequently impacted teeth, followed by their maxillary counterparts and the upper canines. Their retention is influenced by several anatomical and developmental factors, including limited retromolar space, unfavorable angulation, and eruption trajectory—each critical for proper emergence.

**Objective:**

This study examines the association between the eruption or impaction of mandibular third molars and variables such as eruption space, transpalatal width, and eruption angulation.

**Materials and methods:**

Seventy-one mandibular third molars were evaluated using 31 jaw models and 31 orthopantomograms (OPGs). Transpalatal width was measured linearly on the models, while angular data concerning eruption space and direction were derived from the OPGs. Statistical analysis was performed using SPSS (v.25).

**Results:**

Retention was observed in 12.9% of cases. Linear assessments indicated that 51% of the molars were at risk of impaction, contingent on their angulation.

**Conclusions:**

Maxillary constriction significantly increases the risk of mandibular third molar retention. Linear measurements proved more reliable than angular metrics in estimating eruption space. Thus, for diagnosing transverse maxillary discrepancies, linear transpalatal width measurements are preferred over non-metric evaluations.

## 1 Introduction

The third molars (3M), commonly known as “wisdom teeth”, are the last to erupt, typically between 18 and 24. Their retention rate is approximately 98%, with 78% corresponding to the lower third molars (1–3). A recent systematic review and meta-analysis by Pinto et al. (4), covering 98 studies and 183.828 subjects worldwide, reported a pooled prevalence of impacted third molars at 36.9% per subject and 46.4% per tooth. The study also identified demographic predictors, including a higher prevalence in females than males and a greater incidence of mandibular vs. maxillary impactions, underscoring the global clinical importance of this condition.

Retention of the lower 3M is primarily associated with limited space in the mandibular bone (5–7). This retention may occur asymptomatically or be accompanied by discomfort due to complications such as inflammation and infections in the soft tissues of the oral cavity, including pericoronitis and periodontal disease. Complications affecting deeper structures are often related to dental caries adjacent to the retained molars (8–10). Other recognised pathologies linked to lower 3M retention, such as root resorption, cysts, tumours, and mandibular fractures, frequently cause pain, impair masticatory function, and reduce the quality of life of affected individuals (5, 10–12).

Recent studies have investigated the genetic expression changes following surgical extraction of impacted third molars to better understand postoperative complications. Zhou et al. (13) identified 555 differentially expressed genes in gingival tissues, including those involved in immune response and bone mineralization. These findings offer valuable insights for improving postoperative management, personalizing treatments, and developing novel therapies to minimize inflammation and enhance healing after third molar surgery. Furthermore, Motoc et al. (14) highlighted the significant influence of demographic and biological factors such as age, gender, body mass index, diet, and salivary pH on the prevalence of periodontal pathogenic bacteria in children and adolescents, emphasizing the multifactorial nature of oral health and its potential implications for third molar pathology.

Historically, guidelines and recommendations regarding the extraction vs. retention of mandibular third molars have evolved under the influence of institutions such as the Royal College of Surgeons of England and the National Institutes of Health in the United States, as well as regional bodies like the National Institute for Health and Care Excellence (15). Despite these efforts, the surgical removal of third molars remains controversial due to insufficient evidence supporting routine extraction, particularly in asymptomatic and partially erupted cases. Moreover, the increased incidence of distal surface caries (DSC) in second molars adjacent to impacted third molars has been a focal point of clinical concern and guideline formulation over the past two decades.

The size and morphology of the dental arches, along with the dimensions of the mandibular bone, have been studied to predict lower third molar eruption (16). Although various studies have validated the use of linear and angular measurements to assess the likelihood of lower third molar eruption (17–19), the relationship between trans palatal width, available eruption space (ED), and the degree of angular deviation in the eruption direction of the lower third molars remains unclear.

### 1.1 Conceptualisation and background

The eruption of the lower third molar (3M) begins with its calcification around the age of 9 and typically concludes between the ages of 20 and 24. This eruption process is influenced by various factors, including race, diet, genetics, insufficient retromolar space, and specific patterns in the growth of the mandibular ramus, all of which may affect the possibility of eruption (20–23). A lack of retromolar space in the mandible, combined with a marked degree of angulation (i.e., the inclination of the eruption axis), may result in impaction against the second molar, thereby causing the third molar to be deflected towards the lingual cortex of the mandible (20).

**Figure 1:**
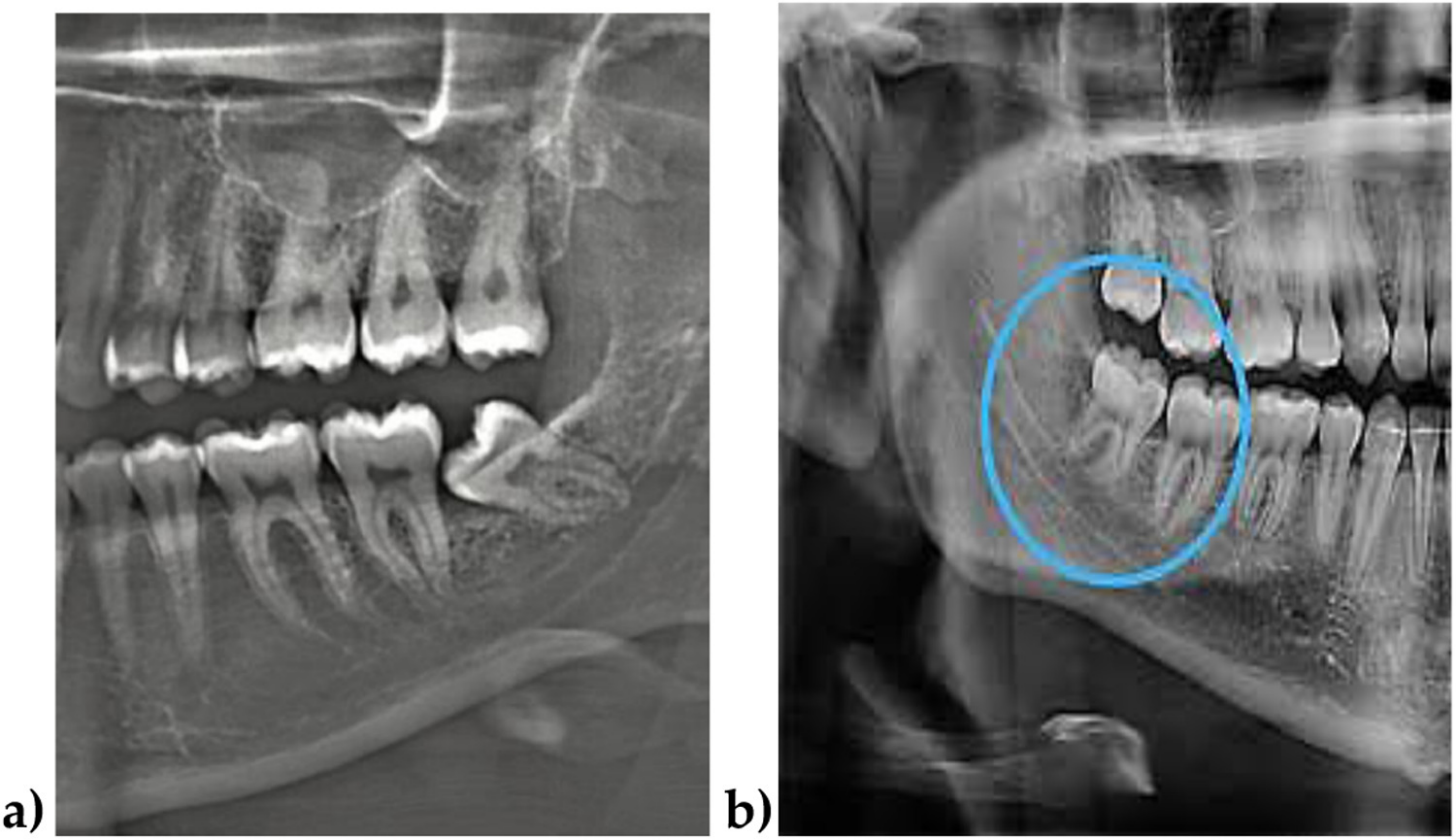
**(a)** Case with no space for the lower third molar eruption due to a lack of mandibular growth. **(b)** Case with space available for eruption of the third molar due to mandibular growth. Source: **(a)**: The authors; **(b)** Dr. Daniel Sepulveda.

When analysing the aetiological factors contributing to lower 3M retention (24–26), highlight the importance of mandibular bone growth direction—specifically, the spatial relationship between the anterior border of the mandibular ramus and the distal surface of the lower second molar—as a determinant of available space for third molar eruption. Horizontal mandibular growth, as opposed to vertical growth, tends to generate greater space for eruption, thereby reducing the likelihood of lower 3M retention (Figure 1).

Several authors agree that a combination of factors determines the etiology of lower third molar retention. These include bone resorption at the anterior border of the mandibular ramus, increased inclination in the direction of growth, and mesial displacement of the teeth. These factors are crucial in determining the available space and are related to the probability of a successful eruption of the lower third molar. Collante & Lewintre (27), Bareiro & Duarte (24), Puyen (25), and Rodríguez del Toro et al. (26) also maintain that if half of the third molar is within the mandibular bone ramus, the probability of eruption is 50%.

There are several classifications for retained third molars, all of which coincide in evaluating their position and relationship to the ascending ramus of the mandible, the retromolar space available for eruption, the angle of eruption, and the tissue coverage over the retained lower third molar. These classifications are fundamental tools for oral surgeons, as they facilitate an accurate clinical diagnosis and surgical treatment planning, allowing them to predict the extraction difficulty and anticipate possible complications.

Among the most commonly used classifications for third molar (3M) retention are those based on the third molar's position relative to the second molar's longitudinal axis. These classifications consider various factors, such as the depth of 3M impaction, its relationship to the lower second molar, the mesiodistal diameter of the retained 3M crown, and the distance between the lower second molar and the anterior border of the mandibular ramus. They are primarily based on three main factors: the depth and direction of the eruption of the third molar, the number, direction, and morphology of its roots, along with two complementary factors: its relationship to the inferior dental canal and the second molar. All these classifications have proven valuable and reliable tools (6, 7, 28).

**Figure 2:**
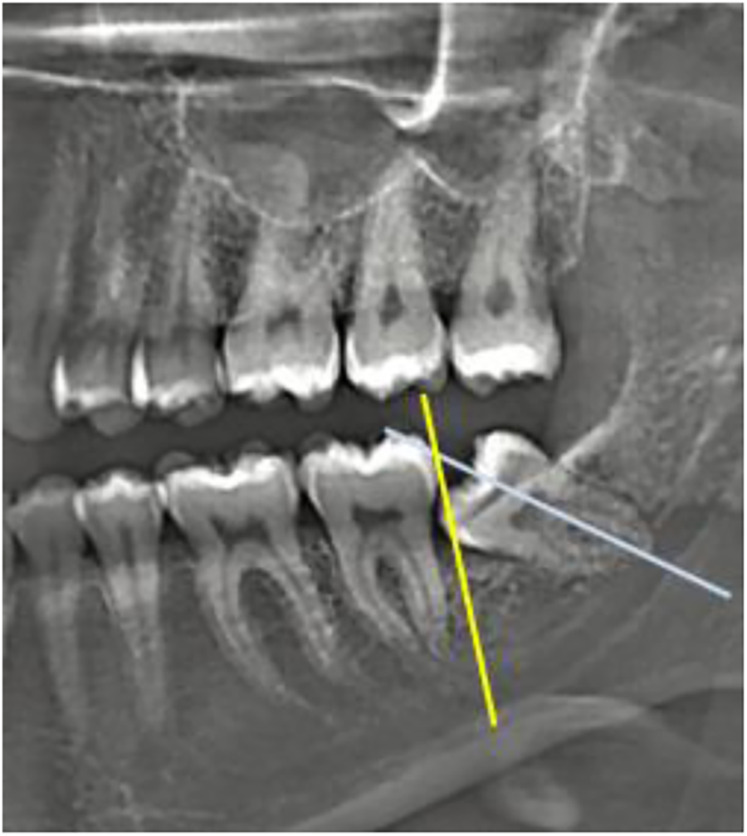
The third molar region will include. Source: The authors.

Within the clinical assessment and evolution of the 3M retention process, the analysis of radiographic records, such as lateral cephalic radiographs and orthopantomographs (OPG), should consider aspects such as the depth and inclination of the tooth, its relation to the mandibular ramus, root characteristics, and ED (17–19) (Figure 2).

A recent review study on third molar (3M) eruption prediction highlighted that, among 2,78 patients assessed at a young age, early evaluation reduced the need for invasive treatments, such as surgical extraction in adulthood, and minimised the risk of associated oral diseases, including pericoronitis, infections, and dental caries in the molars adjacent to the retained 3M. This had a positive impact on the long-term oral and general health of children and adolescents (10).

To predict the likelihood of lower 3M eruption, several studies have emphasised the importance of evaluating factors such as the available eruption distance (ED), crown size, and changes in eruption angulation (18, 19). For this purpose, different analytical methods have been developed, involving both linear measurements of the eruption space and angular measurements of the eruption inclination of the lower 3M, using lateral cephalometric radiographs and orthopantomographs (OPG).

Leon (29) and Mummolo et al. (19), conducted studies to predict 3M eruptions based on linear ED measurements. To do this, they traced the occlusal plane. They established two perpendicular lines and two tangents—one to the distal surface of the lower second molar and the other to the anterior edge of the ascending ramus of the mandible. The linear distance between these tangents was termed the available space (AB), while the mesiodistal width of the third molar crown was defined as CD.

These studies agree that when the mesiodistal width of the 3M crown (CD) is less than the available space (AB), the probability of eruption is high. Conversely, when CD exceeds AB, retention becomes more likely. Furthermore, it has been established that eruption is highly probable when the ratio between these two linear measurements (AB/CD) is equal to or greater than 1.

**Figure 3:**
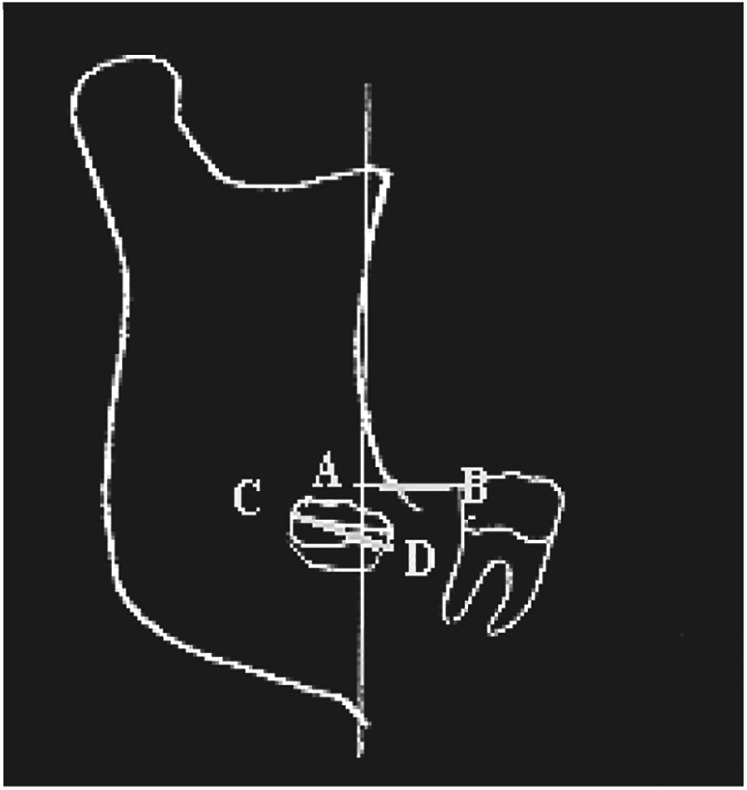
3M eruption probability. Source: Quiros & Palma (18).

On the other hand, if this ratio is less than 1, the probability of eruption is significantly reduced or even absent (Figure 3).

One of the most widely used analytical methods to predict third molar (3M) eruption using linear measurements is the approach proposed by Ganns in 1993. This method determines the available eruption distance (ED). It compares it with the mesiodistal width (MD) of the 3M using the following formula*: X* *=* *ED/mesiodistal width of the lower 3M,* where *X* represents the probability of eruption and the space available for lower 3M eruption. According to this analysis, when *X* ≤ 0.7 mm, the eruption of the lower third molar is unlikely; when X is between 0.71 mm and 0.99 mm, the partial eruption is expected; and when X > 1.0 mm, the full eruption is likely.

Other studies have used linear measurements of ED by applying cephalometric landmarks on lateral cephalometric radiographs. Sánchez (30) and Rodríguez et al. (31), employed the cephalometric point *Xi*, representing the centre of the mandibular ramus, to measure the distance to the distal surface of the second molar, using the occlusal plane as a reference. Their results showed that an ED ranging from 21 mm–29 mm corresponds to partial eruption, whereas distances of 30 mm or more indicate complete eruption of the lower 3M.

In a different approach, Verma et al. (32), Ericsson (33), and Romero et al. (34) combined linear and angular measurements using lateral cephalometric radiographs and orthopantomograms (OPGs) to predict third molar (3M) eruption. Their analysis included linear parameters such as the mesiodistal width of the third molar, the distance between the anterior border of the ascending ramus and the distal surface of the second molar, and the distance from the cephalometric point Xi to the distal surface of the second molar. They also measured the angle of inclination of the third molar relative to its apical base.

**Figure 4:**
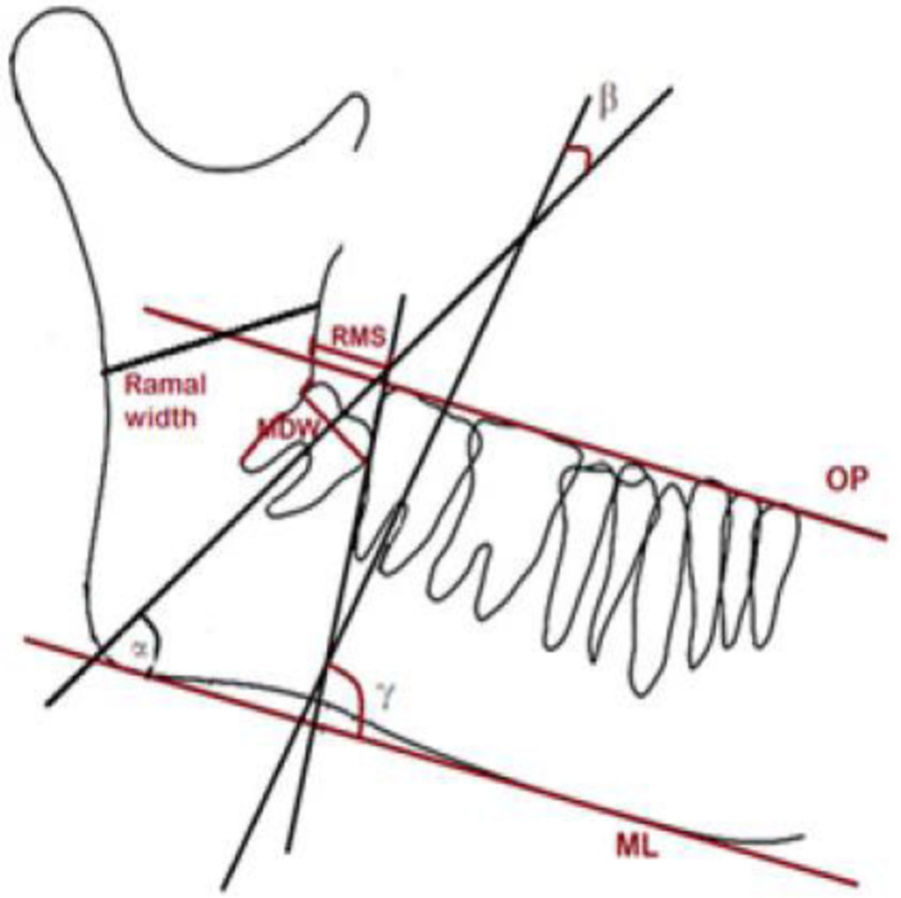
3M eruption potential. Source: Kaur et al. (35).

In a related study, Kaur et al. (35) indicated that three variables must be considered to reliably predict lower third molar eruption: (a) the linear distance from the distal surface of the second molar to the anterior border of the ascending ramus, which should not be less than 14 mm; (b) the distance from cephalometric point Xi to the distal surface of the second molar, which should be at least 35 mm; and (c) the direction of eruption, measured as the inclination of the third molar relative to the occlusal plane, which should be at least 40°, to ensure a favourable eruption path. These findings are illustrated in Figure 4.

**Figure 5:**
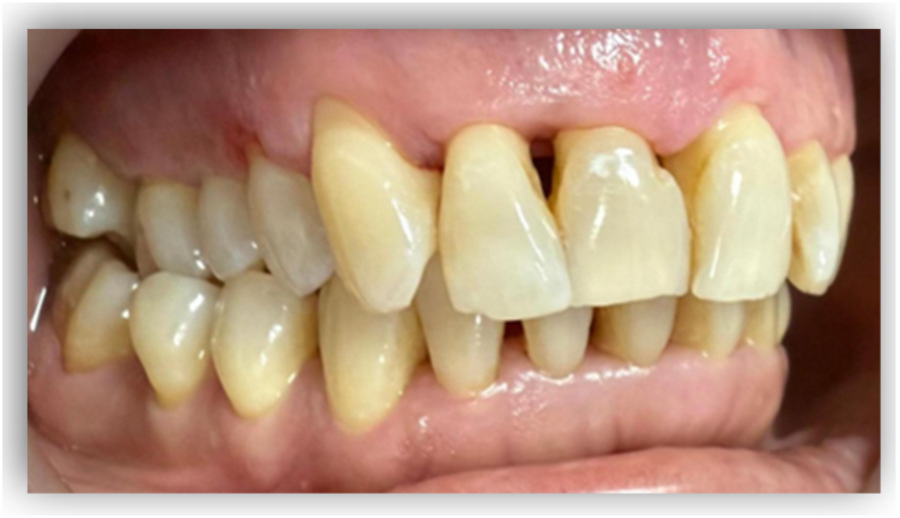
Unilateral crossbite. Source: The authors.

**Figure 6:**
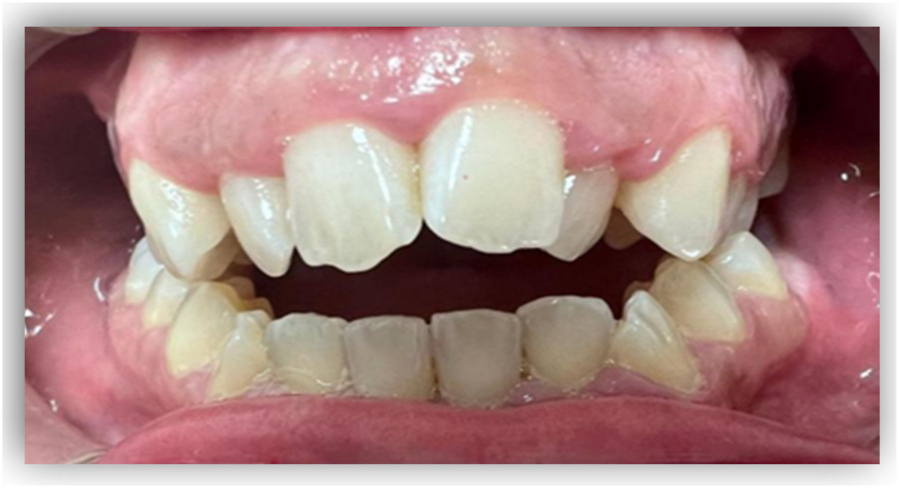
Bilateral crossbite. Source: The authors.

Several studies have identified issues related to transpalatal width in the context of maxillary growth and development, which have been described as transverse malocclusions. These are typically classified as unilateral crossbites (UCD) and bilateral crossbites (BCM) (Figures 5, 6).

To determine transpalatal width (36, 37), measured the distance between canines, premolars, and homologous molars on both sides, using as a reference the most vestibular point of the clinical crown of the canines. They established standard measurements of 30.96 ± 1.8 mm between the first premolars, 39.8 ± 2 mm between the second premolars, and 54.36 ± 2.1 mm between the molars. In addition, they measured the cusp-to-cusp distance of the canines, reporting a normative value of 25.3 ± 1.6 mm.

In a complementary study on dental arch development and size, Mayoral & Mayoral (38) and Mosquera (39) proposed one of the most reliable methods for assessing dental arch dimensions and transpalatal width. These authors measured the transpalatal width by recording the distances between the canines, premolars, and molars, using the central fossae of the first and second premolars as reference points, and the homologous first molars on the right and left sides. They established standard values of 35 mm for the distance between the first premolars, 41 mm for the second premolars, and 47 mm for the first molars. More recent studies have adopted these same parameters and measurements in determining transpalatal widths, including the works of Narciandi et al. (40), Rodrígue del Toro et al., (26), and Lozano Villegas (41).

Having reviewed the background of 3M retention and the prediction of its eruption, this study examined the relationship between transpalatal width and the space available for eruption or retention of the lower third molar by analysing linear and angular measurements in 71 molars from 31 patients. The aim was to understand how these variables correlate and to assess their impact on lower 3M retention. The findings of this research, which explore the relationship between jaw dimensions and third molar retention, may be highly relevant for diagnosis, planning, and preventive treatment in dental practice, thereby contributing to improved oral and overall health outcomes for patients.

### 1.2 Objectives

- Analyse the correlation between lower third molar retention/eruption and predictive variables to determine the relationship between lower third molar retention and/or eruption and various predictive factors, including linear measurements of available mandibular space, maxillary transverse width, and angular parameters influencing eruption direction.
- Assess non-metric indicators of maxillary compression regarding molar retention/eruption: Examine non-metric signs of maxillary compression, such as a narrow or ogival palate, posterior crossbite, protrusion, and dental crowding, to evaluate their potential association with lower third molar retention or eruption.

## 2 Materials and methods

### 2.1 Study design

The study was conducted by the Declaration of Helsinki and was approved by the Ethics Committee of CEIBA, University of La Laguna (Protocol code 2,023–3,337). This descriptive study examined the characteristics of lower third molars through linear and angular measurements obtained from OPGs and study models. The analysis integrated radiographic images and plaster models of the jaws to evaluate key anatomical variables associated with third molar retention and eruption.

### 2.2 Sample

The study comprised thirty-one patients evaluated using thirty-one panoramic radiographs, thirty-one study models, and seventy-one radiographic images of lower third molars.

The sample distribution, categorized by sex and age, is presented in Table 1.

**Table 1:** Distribution of the sample according to sex and age (*n* = 31).

Variables	Levels	*N*	%
Sex	Female	16	51,6
	Male	15	48.4
Total		31	100.0
Age	Minimum–Maximum	Meam	SD
(Years)	11–19	14.9	2.2

### 2.3 Sample selection criteria

Patients were not randomly selected, but had to meet strict inclusion criteria to ensure the homogeneity of the sample and the validity of the analyses performed. Individuals between 11 and 19 years of age with permanent dentition only, fully developed roots in the lower second molars, and no history of previous dental extractions or orthodontic treatments that could alter the morphology or space available in the mandible were chosen. This selection was justified because the study aimed to analyse the relationship between space available for eruption and retention of lower third molars under representative clinical conditions, minimising the interference of external factors or previous treatments.

In addition, patients with dental anomalies, oral diseases, or radiographs of insufficient quality to ensure the accuracy of linear and angular measurements were excluded. This methodological strategy was necessary so that the results were primarily attributable to the patient's anatomical and functional characteristics, ensuring the reliability and validity of the predictions of lower third molar eruption or retention.

This was a retrospective and exploratory study. Therefore, no formal sample size calculation was conducted. The sample was composed of clinical records, study models, and panoramic radiographs from patients who visited a private dental clinic in Plasencia, Spain, over a four-year period. Participants were selected through non-probabilistic, convenience sampling, and only those who met the inclusion criteria were considered. This approach ensured access to high-quality diagnostic material suitable for evaluating third molar eruption and retention.

### 2.4 Procedure and data collection

Participants or their legal representatives, in the case of minors under 18 years old, provided informed consent to allow access to medical records, personal and clinical data, panoramic radiographs, and study models. The study was conducted following the guidelines of the Declaration of Helsinki.

The topics were extracted from keywords derived from the research question to ensure rigorous data collection and purification. The most frequent keywords in related studies were initially identified to refine the search for these topics.

The most appropriate search equations were formulated from this stage by combining keywords using Boolean operators (AND, OR). To determine the affinity of studies with the objectives of this research, their relevance and suitability were assessed by reading abstracts and the full texts.

#### 2.4.1 Radiographic procedure

Panoramic radiographs were obtained using a CRANEX OME CEPH radiographic unit (Orion Corporation Sonedex, Finland). According to the manufacturer's specifications, the equipment presents a 30% inherent distortion (expansion) due to the mandible's three-dimensional structure.

#### 2.4.2 Sample classification

Cases were classified by dentition type, including 31 panoramic radiographs from 16 female and 15 male patients. All patients exhibited permanent dentition and met the study selection criteria.

#### 2.4.3 Manual measurements

Measurements were manually taken on study models using a Limit brand digital caliper with automatic shut-off function at 300 mm and 150 mm (manufacturing code 29281201, serial number 001027), compliant with ISO 9001:2000 standards. All measurements adhered to established linear and angular criteria.

To ensure the reliability of the measurements, all linear and angular assessments were conducted by a single trained examiner. To assess intraobserver reliability, 25% of the sample was remeasured after a two-week interval under the same conditions. The intra-class correlation coefficient (ICC) was used to evaluate reproducibility, yielding values above 0.90 for all variables, which indicates excellent reliability.

#### 2.4.4 Maxillary width and palatal morphology analysis

The digital caliper measured the maxillary transpalatal width from the central fossa of the first and second upper premolars to their contralateral counterparts. The intermolar distance between the first upper molars was also evaluated. Standard reference values were:

- First premolars: 35 mm
- Second premolars: 41 mm
- First molars: 47 mm

Measurements below these values indicated maxillary compression.

Palatal morphology was analysed on the models to diagnose maxillary compression or narrowing and to determine the presence or absence of an ogival (V-shaped) palate. Palatal morphology was classified as either ogival (V-shaped) or normal (U-shaped).

#### 2.4.5 Anterior teeth protrusion

The protrusion of the upper anterior teeth was evaluated by measuring the distance (in mm) from the upper incisors' incisal edge to the lower incisors' vestibular surface, with protrusion classified according to the measured distance.

#### 2.4.6 Panoramic radiograph analysis: space and angulation for eruption

All panoramic radiographs were obtained using the same equipment: CRANEX OME CEPH (Orion Corporation Sonedex, Finland). Exposure parameters ranged from 60–90 kV, 4–15 mA, and 10–20 s, depending on individual patient characteristics. The device underwent regular calibration, and geometric accuracy was verified using a phantom model with known dimensions.

Panoramic imaging introduces an estimated 30% horizontal expansion, particularly noticeable in the anterior mandibular region due to its curved anatomy. However, in the posterior (molar) region, this distortion is minimal, thus allowing reliable linear and angular measurements of the third molar.

To assess the available space for eruption or retention of the lower third molar, a line representing the occlusal plane was drawn on the panoramic radiograph. A perpendicular reference line was projected from the most distal point of the crown of the lower second molar. The available space (line AB) was measured from this point to its intersection with the anterior border of the mandibular ramus. Additionally, the mesiodistal width of the third molar crown (line CD) was recorded.

The eruption index (X) was calculated to estimate the probability of lower third molar eruption using the following formula:

$$X=\mathrm{EdMDX}=\frac{\mathrm{Ed}}{\mathrm{MD}}X=\mathrm{MDEd}$$

where:

- Ed (or AB) is the available space (mm), defined as the linear distance from point A (distal surface of the second molar) to point B (intersection of the occlusal plane with the anterior border of the mandibular ramus).
- MD (or CD) is the mesiodistal crown width of the third molar (mm), measured from point C (distal surface of the third molar crown) to point D (mesial surface of the same crown).
- X represents the eruption index, indicating the probability of eruption.
- Interpretation of the index:
- X ≤ 1.0 (0–0.99 mm): No possibility or partial eruption.
- X > 1.0 (≥1 mm): Higher probability of complete eruption.

To summarise these aspects, a summary of the variables considered for the study is presented in Table 2.

**Table 2:** Conceptualization and description of the variables of the study.

Variable	Conceptualization	Dimension	Type of Measure	Scale	Category	Categorization
Space available for eruption of lower third molars	Difference between the distance measured in mm from the anterior border of the ascending ramus of the mandible to the distal surface of the second lower molar.	MD in mm lower third molar crown.	Lower third molar MD width.	Ratio	0–99	<1 non-eruptive
					1- higher	>1 eruptive
		Ganns Index [27]	Available space in mm (AB)/width MD (CD) of the lower third molar	Ratio	0,5–0,99	<1 non-eruptive
					1- higher	>1 eruptive
Direction or angulation of eruption of lower third molars	The angle formed by the axis of the lower third molar in the direction of its apex and the perpendicular to the major axis of the lower second molar, mean in degrees.	Index Quiroz and Palma [15]	Lower third molar inclination degrees	Ratio	10–20 degrees	<40 degree angle
					20–29 degrees	
					30–39 degrees	
						>40 degree angle
					>40 degrees	
Transverse diameter of the upper jaw (Bone)	Linear distance from the central fossa of the first premolars, second premolars, and upper first molars to their contralateral counterpart	Mayoral and Mayoral Index[36]	Measurement of interpremolar, first premolars, second premolars and intermolars from the central fossa of each of these teeth with their collateral counterparts.	Ratio	1° Premolar	<35 compression
					25–30 mm	
						>35 without compression
					31–35 mm	
					36- higher	
					2° Premolar	<41 compression
					31–35 mm	
						>41 without compression
					36- 40 mm	
					41- higher	
					1° Molar	<47 compression
					37–41 mm	
						>47 without compression
					42–46 mm	
					47- higher	
Protrusion of upper anterior teeth	The location of the central incisors in the upper alveolus exceeds the horizontal space in centric occlusion between the palatal side of the upper incisor group and the vestibular side of the lower anterior teeth above 2 mm.		Data observed in study models	Nominal		Protrusion
						No protrusion
	Alteration of the hard palate with elevation of its central part with a marked arching of the sides. Draw a V-shaped or ogival figure.		Data observed in the study models	Nominal		With Ogival Palate
						Without Ogival Palate
Posterior upper crossbite	Ocurre cuando las cúspides vestibulares de los premolares y molares superiores ocluyen en las fosas de los premolares y molares superiores	Ogival or narrow palate	Data observed in the study models	Nominal		With crossbite
						No crossbite
Gender	Organic condition according to the sex organ		Data observed in the medical history and x-rays	Nominal		Male
						Female

## 3 Data processing

### 3.1 Analysis approach

The data collected from the study models' observations and panoramic radiographs, which were systematically recorded in specially designed tables, underwent comprehensive analysis. This analysis utilised the Statistical Package for the Social Sciences (SPSS), version 25.

### 3.2 Statistical methods

A range of statistical methods was employed to analyse the data rigorously. Descriptive statistics were used to summarise and interpret the data succinctly. Contingency tables facilitated the exploration of relationships between variables, while the Chi-square test assessed associations and identified potential patterns. This test is instrumental in examining differences in the distributions of categorical variables. Risk estimation techniques were also applied to evaluate the probability of specific outcomes, offering insight into potential risk factors or correlations. Lastly, the COR test was utilized to determine the strength and direction of the relationship between two variables.

## 4 Results

### 4.1 Measurements of upper jaw width in plaster models

The initial phase of the analysis focused on determining the transverse width of the upper jaw using plaster models, following established measurements (38).

The results revealed the following average distances: The distance for the upper first premolars averaged 21.8 mm (SD 2.9). The distance for the upper second premolars was 36.7 mm (SD 3). The upper intermolar distance averaged 43.12 mm (SD 3.2).

These measurements offer a comprehensive overview of the transverse dimensions of the upper jaw in the studied sample (Table 3).

**Table 3:** Results of upper jaw width measurements (mm).

Upper jaw width	Tooth	X	SD	Minimum Value	Maximum Value
Upper jaw width (mm)	1st Premolar	32.5	2.9	27.8	40.9
	2nd Premolar	36.7	3.0	30.0	43.0
	1rd Upper Molar	43.1	3.2	31.9	48.0

The results of the upper jaw's transverse skeletal malocclusion were obtained and presented in Table 3. Upon analyzing the variables indicative of maxillary compression (39, 41–43) and considering additional factors such as posterior crossbite, the presence of an ogival palate, and dental protrusion to assess transpalatal and sagittal width, it was found that 83.9% of the upper first premolars exhibited transverse maxillary compression. Moreover, measurements of the second premolars and intermolar distances revealed that 87.1% of the patients displayed maxillary compression. Analysis of the other diagnostic variables for compression revealed that 35.5% of patients had a posterior crossbite, 87.1% exhibited an ogival palate, and 54.8% presented with dental protrusion (Table 4).

**Table 4:** Diagnosis of maxillary transverse malocclusion by compression or transverse narrowing of the maxilla according to linear measurements of transpalatal width and other non-metric indicators of transverse malocclusion.

Transpalatine width	Tooth/Indicator	Condition/Category	*N*	%
Inter premolar linear measurement	1st Upper Premolar	Compression	26	83.9
		No Compression	5	16.1
	2nd Upper Premolar	Compression	27	87.1
		No Compression	4	12,9
Inter molar linear measurement	1st Upper Premolar	Compression	27	87.1
		No Compression	4	12.9
	Cross Bite	Yes	11	35.5
	Posterior	No	20	64.5
Other non-metric indicators of upper jaw compression	Ogival Palate	Yes	27	87.1
		No	4	12.9
	Upper dental protrusion	Yes	17	54.8
		No	14	45.2

A new variable was introduced to assess maxillary compression or narrowing based on the three transpalatal width measurements. If at least one of the three values indicated a positive result, the patient was diagnosed with transpalatal compression or narrowing of the maxilla. Conversely, if all the values were negative, indicating the absence of narrowing, the patient was classified “without compression.” A summary of the measurements regarding the presence of compression or transpalatal narrowing is presented in Table 5. It was found that 90.3% of the cases exhibited compression, while 9.7% did not have compression of the upper jaw.

**Table 5:** Summary measure of compression or transverse narrowing of the maxilla.

Assessment category	Condition	*N*	%
Transverse measurement or transverse compression	Compression	28	90.3
	No Compression	3	9.7
Total		31	100

If at least one of the three diagnostic values proposed by Mayoral & Mayoral (38) is positive, the case will be considered compression.

The available space (ED) for the eruption of the third molars was measured using panoramic radiographs. Linear measurements of eruption space (AB) and crown width (CD) were employed to predict molar retention, while angular measurements assessed the direction of eruption of the lower third molars. The AB space measurements averaged 21.8 mm ± 4.3 for the right molars and 21.7 mm ± 3 for the left molars. For the CD measurements, the mean value for the lower right third molar was 15.4 mm ± 3.2, while for the left, it was 13.7 mm ± 9.9. The eruption direction inclination was 38.2° ± 16.8 for the right molars and 31.2° ± 10.8 for the left lower third molars (Table 6).

**Table 6:** The results of the measurements of eruption space (ED), crown width (CD), and eruption inclination of the lower third molar are as follows.

Measurement	Mean	SD	Minimum value	Maximum value
Distance AB (R)	21.8	4.3	12.8	30.0
Distance AB (L)	21.7	3.8	15.2	28.5
Width CD (R)	15.4	3.2	12.7	29.0
Width CD (L)	13.7	9.9	1.0	16.8
Inclination (R)	38.2	16.8	3.5	98.0
Inclination (L)	31.2	10.8	3.8	51.0

L, left; R, right.

The results also encompassed predictive variables for lower third molar retention, particularly the ratio between available space (AB) and mesiodistal crown width (CD), as well as the eruption angle, which were used to estimate the eruption prognosis. It was observed that, according to eruption space measurements, 13.3% of the patients were likely to retain their lower third molars. Conversely, 50% were expected to retain the molars using molar angulation or inclination measurements, while the remaining 50% were predicted to experience their eruption. These findings suggest that linear measurements of available eruption space may underestimate the diagnosis, whereas angular inclination measurements may overestimate it (Table 7).

**Table 7:** Prediction of lower third molar eruption with linear and angular method.

Method	Tooth position	Condition	*N*	%
Linear method	Right molar	Retention (R)	4	12.9
		Eruption (R)	26	83.9
	Left Molar	Retention (L)	1	3.2
		Eruption (L)	25	80.6
Angular method	Right molar	Retention (R)	15	48.4
		Eruption (R)	15	48.4
	Left Molar	Retention (L)	7	22.6
		Eruption (L)	19	61.3

L, left; R, right.

Continuing the analysis of retention predictors, linear and angular measurements were employed without distinguishing between right and left molars, yielding the results shown in Table 8.

**Table 8:** Summary measure of eruption/retention prediction based on linear and angular measurements.

Method	Condition	*N*	%
Linear method	Retention	4	12.9
	Eruption	27	87.1
Angular method	Retention	16	51.6
	Eruption	15	48.4

A case was considered retained if at least one lower-third molar head was retained.

Using the linear measurement method (AB/CD ratio), retention was estimated in 12.9% of patients, whereas the angular method (based on inclination <40°) predicted retention in 51%. These values correspond to different diagnostic indicators, applied independently to the same cohort. The angular method tends to overestimate retention, while the linear approach underestimates it (Table 9).

**Table 9:** Relationship between the predictive methods of linear space-available and angular measures for retention/eruption of lower third molars as a function of sex.

Method	Sex	*N*	Retention (%)	*N*	Eruption (%)
Linear method	Female	2	12.5	14	87.5
	Male	2	13.3	13	86.7
Total linear method		4	12.9	27	87.1
Angular method	Female	9	56.3	7	43.8
	Male	7	46.7	8	53.3
Total angular method		16	51.6	15	48.4

Regarding the interaction between gender and third molar eruption prognosis, the analysis showed that, based on linear measurements, 12.5% of women were estimated to have a poor prognosis for eruption (i.e., predicted retention), compared to 13.3% of men. Conversely, favorable eruption was projected in 87.5% of women and 86.7% of men, with no significant gender differences (*p* = 0.72).

The final analysis compared both predictive methods to determine the more accurate predictor of lower third molar retention. The comparison, conducted using the COR (C-statistic) curve, revealed a higher value for the linear measurement method (COR: 0.429) compared to the angular method (COR: 0.399). The area beneath the curve for the linear method was graphically larger, suggesting its superior predictive ability for both retention presence (sensitivity) and absence (specificity) (see Table 9).

Additionally, inter-method comparisons were performed using the Kappa statistical test to assess agreement between the linear and angular measurement methods. The resulting Kappa value of 0.244 indicated a low level of agreement in retention diagnosis. Moreover, a *p*-value of 0.038 suggested a statistically significant relationship between them (Table 10).

**Table 10:** Comparison between linear and angular methods. COR and KAPPA curve test.

Test	Result	
Sig. level		0.038
Kappa measurement		0.244
COR Curve	Linear measurements	0.429
	Angle measurements	0.349

Based on the compression variable assessed through linear measurements, the predictive values for retention or eruption were not statistically significant. However, in evaluating retention risk, patients with decreased transpalatal width, as measured by the linear method, exhibited a higher risk (RR: 1.13 for narrowing or compression).

Similar results were observed in the angular measurement analysis, where maxillary compression was associated with an increased retention risk (RR: 1.08) compared to RR: 0.46 in non-compressed cases. Additionally, posterior crossbite, assessed using the linear method, was associated with an elevated retention risk (RR: 2.5) (Table 11).

**Table 11:** Result of the linear indicators of maxillary compression relationship with the retention of the lower third molars.

Prediction type		Linear Retention		Linear Eruption		Retention		Eruption	
Indicator	Condition	N	%	N	%	N	%	N	%
Palate Compression	Compression	4	14.3	24	85.7	15	53.6	13	46.4
	No compression	0	0.0	3	100.0	1	33.3	2	66.7
	Total	4	18.9	27	87.1	16	5.6	15	48.4
Posterior Crossbite	No	1	5.0	19	95.0	9	45.0	11	55.0
	Yes	3	27.3	8	72.7	7	63.6	4	36.4
	Total	4	12.9	27	87.1	16	51.6	15	48.4
Anterosuperior tooth protrusion	No	2	14.3	12	85.7	7	50.0	7	50.0
	Yes	2	11.8	15	88.2	15	88.2	9	52.9
	Total	4	12.9	27	87.1	16	51.6	15	48.4
Ogival Palate	No	4	14.8	23	85.2	15	55.6	12	44.4
	Yes	0	0.0	4	100.0	1	25.0	3	75.0
	Total	4	12.9	27	87.1	16	51.6	15	48.4

Higher retention risk according to the linear measurement method if compression is present (RR: 1.13; 95% CI: 0.9–1.23) and according to the angular measurement method (RR: 1.08; 95% CI: 0.86–1.37). There is a higher risk of retention according to the linear measurements if there is posterior crossbite (RR: 2.53; 95% CI: 1.12–5.69) and according to the angular measurement method (RR: 1.60; 95% CI: 0.60–4.50) the rest of the RR test have no logical tendencies, they have no significance.

## 5 Discussion

The prevalence and management of third molars continue to be a topic of global concern. Meta-analyses report an approximate 37% prevalence of third molars per patient, with higher rates in females and more frequent mandibular impactions (4). These epidemiological insights emphasise the relevance of predictive studies like ours, aimed at identifying factors influencing lower third molar retention and eruption.

Our findings highlight a strong association between reduced transverse maxillary development and the retention of third molars. Specifically, 90.3% of patients presented with maxillary compression and ogival palates, supporting earlier observations by Mayoral & Mayoral (38), González et al. (44), and Rodríguez Del Toro et al. (26). The linear measurements of dental arch width obtained in this study are also consistent with previously reported norms (36, 38), validating the clinical patterns observed.

Early diagnosis has been widely recognized as a cornerstone in the management of third molars, potentially reducing the need for surgical intervention and associated complications (10, 17, 45). Recent molecular advances, such as those by Zhou et al., further enhance our understanding by revealing changes in gene expression in gingival tissues post-extraction, offering novel insights into postoperative care.

Consistent with earlier literature (35, 45), our study confirms that factors such as limited eruption space, molar inclination, and crown size critically affect third molar positioning. The AB/CD ratio proved predictive: values ≥1 indicated a 70% probability of eruption, while ratios <1 significantly lowered this likelihood, mirroring the results of Ganns et al. (46). Eruption inclination angles (average 38.2° and 31.2°) also fell within ranges reported in prior predictive models (19).

A notable finding was the elevated risk of third molar retention in patients with posterior crossbite (RR: 2.5), which aligns with evidence linking vertical growth patterns and the mesial inclination of the third molar crown to an increased retention risk (25–27). These morphological traits are often associated with inadequate mandibular growth and reduced retromolar space, as previously described by Puyen (25), Rodríguez del Toro et al. (26), and Plaza et al. (47). Such anatomical limitations suggest a broader link between third molar retention and skeletal Class II patterns, particularly those with diminished anteroposterior maxillary dimensions (48, 49).

Significantly, while over 80% of our sample showed maxillary compression, this high prevalence limits the generalizability of our findings. The reduced variability in maxillary width may limit the statistical power to detect differences across subgroups. Thus, although a strong correlation was observed, caution should be applied in extending these conclusions to more diverse populations. Future studies should explore these associations in larger and more heterogeneous cohorts.

Lastly, our results support the evolution of clinical approaches to third molar management, advocating for individualized assessment rather than routine extraction. Concerns such as the development of distal surface caries in adjacent second molars remain central to decision-making, reinforcing the importance of early and precise diagnostic evaluation.

## 6 Limitations

Despite the contributions of this study, several limitations should be considered when interpreting the findings. The sample size may not adequately represent the general population, which constrains the generalizability of the results. Furthermore, the cross-sectional design does not allow for the establishment of causal relationships between predictive variables and third molar retention or eruption. Radiographic evaluations, particularly angular and linear measurements, are susceptible to potential measurement errors, which can impact accuracy. Additionally, the study did not control for relevant confounding variables such as genetic predispositions, environmental influences, or coexisting oral conditions that may affect the eruption process. Another important limitation is the lack of longitudinal follow-up, which restricts insight into the progression of third molar development over time.

Moreover, the assessment of transpalatal width was based exclusively on dental measurements obtained from plaster models. Skeletal parameters, such as those evaluated in Andrews' Element III analysis, were not included due to the retrospective nature of the data and the absence of three-dimensional imaging or articulated models. While dental metrics provide useful clinical information, they may not fully reflect underlying skeletal discrepancies. Future studies should consider integrating skeletal assessments to enhance diagnostic precision.

## 7 Conclusions

This study presents key findings relevant to the prediction of lower third molar retention and eruption:

- Maxillary compression was observed in 90.3% of patients, with a strong correlation between compressed or ogival palates and molar retention.
- Linear transpalatal measurements proved reliable in identifying transverse maxillary narrowing, in line with previous research.
- The AB/CD ratio emerged as a useful predictor:
  ○ AB/CD ≥ 1 → ∼70% eruption probability;
  ○ AB/CD < 1 → increased retention likelihood.
- Angular measurements also supported the prediction of eruption direction or retention.
- Higher retention risk was associated with:
  ○ Maxillary compression;
  ○ Posterior crossbite;
  ○ Upper anterior dental protrusion;
  ○ Skeletal Class II patterns and insufficient mandibular growth.
- Posterior crossbite, linked with vertical growth and mesial crown inclination, was a particularly strong indicator of retention risk.

Future studies should be designed with larger and more diverse cohorts to improve external validity. Incorporating longitudinal follow-up would provide valuable data on temporal changes in third molar positioning. Moreover, controlling for potential confounding factors and exploring genetic and systemic determinants would enhance the understanding of the multifactorial nature of molar eruption and retention.

## Data Availability

The raw data supporting the conclusions of this article will be made available by the authors, without undue reservation.
